# Refractory Sexual Arousal Subsequent to Sacral Neuromodulation

**DOI:** 10.1155/2019/7519164

**Published:** 2019-04-02

**Authors:** Dani Zoorob, Amanda S. Deis, Kathryn Lindsay

**Affiliations:** ^1^Department of Obstetrics and Gynecology, University of Toledo, 2142 N. Cove Blvd., Toledo OH 43606, USA; ^2^Department of Anesthesiology and Perioperative Medicine, University of Pittsburg, A-1305 Scaife Hall, 3550 Terrace Street, Pittsburgh, PA 15261, USA; ^3^Department of Obstetrics and Gynecology, University of Oklahoma, 825 NE 10th St, Oklahoma City, OK 73104, USA

## Abstract

**Background:**

Sacral neuromodulation has become a widely used treatment for lower urinary tract symptom and dysfunction. It has been observed to benefit sexual function in the domains of arousal and desire. Studies have yet to report markedly increased arousal symptoms as an adverse effect.

**Case:**

We present the case of a 57-year-old woman who developed symptomatic persistent genital arousal following implantation of a neuromodulator. Despite device reprogramming, a trial of the device being shut off, and eventual device removal, she continued to have residual new-onset undesired genital hyper-arousal symptoms.

**Conclusion:**

Our patient demonstrated markedly increased and persistent arousal symptoms that may be the result of upregulated or alternative activation of sacral nerve pathways. While other case reports describe improvement in persistent genital arousal disorder symptoms through neuromodulation, no studies mention hyperarousal symptoms as an adverse side effect after sacral neuromodulator placement nor persistence despite removal of the implant.

## 1. Introduction

Overactive bladder symptoms are common and affect a considerable number of female patients in the United States [1]. Patients can be managed in several ways, one of which is through sacral neuromodulation (SNM) using the InterStim II™ system (Minneapolis, Minnesota/USA). SNM is an FDA approved non-pharmacological option for the treatment of urgency incontinence [2]. The exact mechanism of action of SNM is unknown but theorized to normalize neural communication between the sacral viscera and the brain, treating symptoms of urgency, frequency, and urinary retention [2, 3]. Studies show that SNM is effective in control of symptoms with a limited side-effect profile [4, 5]. The most common documented side-effects are suspected lead migration (9%), infection (9%), sensation of electrical shock (8%), and undesirable change in voiding function (7%). Reports have also documented SNM use to normalize sexual arousal, however only while the implant is activated [6, 7]. In the current literature, we have not been able to identify other cases reporting new-onset markedly increased and sustained sexual arousal with persistence of the condition despite explant of the sacral neuromodulator.

## 2. Case

The patient is a 57-year-old nulligravid female who presented to clinic with symptoms of urinary urgency incontinence. She had a past medical history of endometriosis, autoimmune hepatitis, cirrhosis, and denied any pertinent mental/psychological history or trauma. Her initial symptoms included urgency, voiding up to 5 times an hour, and nocturia up to five times per night. She had no previous vaginal surgeries. Baseline sexual function evaluation was completed at intake with the Female Sexual Function Index (FSFI) questionnaire where the arousal domain equaled 0.9 (range 0-6, with 6 indicating maximal arousal) (Table 1). Pelvic examination was significant for vaginal atrophy but no notable clitoral or labial abnormalities were visualized. There were no signs of prolapse or pelvic floor musculature hypertonicity. She was asked to keep a voiding diary and then advised to attempt timed voiding upon its completion. She was also started on vaginal estrogen and a generic anticholinergic agent (oxybutynin 10 XL daily). After 4 weeks of treatment, she experienced significant worsening of anticholinergic side-effects and stopped the medication on her own. She was then started on a beta sympathomimetic (mirabegron 25mg daily). After 6 weeks of this therapy, she did not have any subjective improvement in her symptoms. The dose was increased to 50 mg daily and continued for an additional 4 weeks. Again, she did not have an adequate reduction in symptoms and was counseled on third tier treatment options. Ultimately the decision was made to proceed with SNM.

The sacral neuromodulator was implanted per manufacturer instructions after undergoing a peripheral nerve evaluation with >50% improvement in her urgency symptoms. At one-week follow-up, her incisional pain was minimal. She did not require oral analgesics and had reported marked improvement in urinary symptoms consistent with the test phase.

However, near the six-month follow-up appointment, she expressed concerns about persistent arousal symptoms in the vaginal area overall with new onset hypersensitivity localized to the clitoris. She did not have these symptoms prior to or immediately postimplant, but she reported gradual development of arousal symptoms postoperatively over the six-month period. She had not initiated the use of any new medications or therapies during the same time period.

Pelvic examination did not demonstrate engorgement of the clitoris, change from the intake examination, or evidence of hypertonic pelvic floor muscle dysfunction based on digital assessment. To manage her arousal, the four programs that were programmed into the system were alternated with cycling activated. Behavioral modifications were suggested including loose clothing. Upon no change in symptoms, device deactivation was performed. This resulted in no notable improvement of the manifest arousal symptoms. However, her urgency symptoms immediately recurred upon the deactivation. At this point, the plan was to trial a new set of programs to see if her arousal symptoms could be eliminated using different settings. The patient agreed to trial all four new programs, each over at least a 10-day period and assess which one was associated with fewer arousal symptoms. The programming was done at sensory levels. Alternation of pulse width and frequency was performed as well. The patient was instructed to complete a 4-week diary indicating arousal and bladder activity. At follow-up, she had trialed each program and continued to experience sexual arousal symptoms. Her symptoms were present even when the device was turned off and intensified when the machine was on. The symptoms were also present without any clitoral contact by undergarments. It was becoming so bothersome that it was difficult for her to stay asleep at night and creating anxiety. Complete testing of the neuromodulator unit was reperformed with normal values noted for impedance. Reprogramming of the unit with a new set of programs was performed again with subsensory levels used at this point. Additionally, she was offered sexual counseling but it was declined.

One year after placement, she elected for removal of the generator and lead. The generator and lead, intact with tip, were successfully removed with no complications. At the 6-week follow-up after explant, she had recurrence of urinary urgency symptoms with persistence of hyper-arousability. One year later, she continues to be sexually active with mild discomfort due to vaginal atrophy (as noted on the pain domain in the FSFI). She reports that the clitoris remains hypersensitive with persistence of the arousal symptoms, although moderately improved from prior to SNM. At the follow-up visits, no changes in medications or new medical diagnoses were reported when compared to prior visits. Furthermore, she denied any new stressors or change in her personal life regarding the relationship with her husband.

## 3. Discussion

In this report, we provide an account of a patient experiencing new-onset symptoms of persistent sexual arousal following placement and removal of a sacral neuromodulator. The diagnosis of persistent genital disorder is made when patients have extended arousal or sexual response lasting for hours or days, may not resolve despite orgasm, the symptoms are considered intrusive and undesired, elicited by both sexual triggers and non-sexual stimuli, and the feelings cause distress and fear [8]. In the patient reported, the symptoms were not resolved with device reprogramming, a trial of the device being shut off, or even device removal. These symptoms were absent prior to the implantation, thus raising the query of whether the symptoms arose from activation of sacral nerves associated with sexual function once the reflex pathways were activated. Medline search between 1980 and 2019 using either terms persistent genital arousal or hyper-arousal concurrent with SNM demonstrated two articles that use SNM specifically for reduction of arousal. However, none in the literature discuss resultant persistent arousal.

The exact nervous system anatomy per se that is involved with female sexual arousal is unknown but a large component is thought to be a product of the spinal cord reflexes involving the pudendal and sacral nerves [9, 10]. A known spinal reflex involves contraction of the pelvic floor muscles, associated with orgasm following stimulation of the S2,3,4 segments of the sacral plexus [9]. Additionally, the clitoris has significant innervation emanating from the branches of the pudendal nerve [11].

After use of SNM, it is possible that our patient experienced an increase neural input that activated these nerves leading to the augmented tactile sensations of arousal she experienced. Parnell et al. discussed such input and documented measurable electrophysiological changes in the pudendal nerve after placement of the SNM device [12]. Their study measured improvement in pudendal nerve function and statistically significant improvements in sexual function scores were noted in the desire domain of the FSFI. Similar to the increase in FSFI scores in our patient, SNM has been reported to help improve the FSFI scores to the non-dysfunctional level [6, 7, 13] (Table 1). A systematic review of nine articles investigating the role of SNM on sexual function showed improvement in at least one female sexual function domain, but no reports of hyperarousal were mentioned [14]. Persistence of symptoms, if the explant was performed, was not cited either. Alternatively, some SNM accounts report its use to address persistent genital arousal; however, these do not report on the permanency or long-term arousal effects [15]. Malouf et al. alluded to the subject of permanency of neuro-modeling [16]. They reported that the ‘beneficial effect is related to the stimulation and is dependent on continued stimulation. Stimulation does not induce any permanent change in function, at least not in the duration of treatment we observed'. The effect they assessed related to incontinence, which is rapidly and easily measurable. However, when it comes to arousal, the duration of our patient's symptoms lasted for at least a year based on her follow-up, despite device removal, suggesting a potential remodeling of the neural circuits involved. The FSFI noted persistent elevation of the arousal domain despite explant and the working theory is that neuromodulation effects could have persisted due to permanent activation of feedback pathways that the stimulation triggers. Development of hypertonicity of the pelvic floor musculature is another consideration that could explain the hyper-arousability upon wearing tight clothing. However in this patient specifically, pelvic examination did not demonstrate high tone pelvic floor disorder on preoperatively or postoperatively. Ideally, MRI imaging of the sacral region should be performed to assess for Tarlov cysts as well as anomalies in vasculature in the clitoral region as these are a part of the differential diagnosis explaining development of such symptomatology. Tarlov cysts may present at the level of S1 to S4/5 and are often correlated with birth trauma. The presence of these structures may increase pelvic floor tonicity and neuromodulation may have unmasked their effect resulting in perceived sexual sensitivity in patients. Knowing that the patient was nulligravid and had no prior history of pelvic trauma (except for the implant), and since she already had the implant in place, assessment for pelvic abnormalities by the MRI technique was deferred. Although not ideal, CT imaging was offered but the patient did not desire to undergo further imaging. Keeping in mind that effects may not necessarily be limited to physical causes, the mental status was assessed at the initial intake and during the workup prior to the removal of the implant. It is known that many psychotropic medications have significant sexual side-effects including but not limited to impact on libido and anorgasmia. Thus, the assessment addressed initiation or change in medications as well as new diagnoses of medical conditions. At intake and the visit prior to removal, the patient denied history of trauma or the use of antidepressants/mood stabilizers.

A major limitation to this report includes absence of a formal psychological evaluation using a validated tool targeting stressors, traumatic conditions, as well as mental health status. Furthermore, nulliparity and lack of pelvic trauma do not preclude the need for imaging that could unveil a structural abnormality that was exposed by the SNM implant. Thus the lack of these evaluations could still suggest reasons for the persistent arousal this patient developed.

Although achieving arousal may be considered a positive effect, the resultant persistent hyper-arousability that this patient encountered had a significant negative impact on her quality of life. Thus, the possibility of discussing a potential sexual impact as well as the expected urinary improvement may need to be discussed with potential candidates when consenting for the procedure of sacral neuromodulation.

## Figures and Tables

**Table 1:** FSFI scores prior to implant, 6 months following the implant, and 12 months following the explant.

Domains	Pre-Procedure	Post-Implant^∗^	Post-Explant^∗∗^

Desire	1.2	2.4	1.2

Arousal	0.9	6	4.5

Lubrication	0	2.4	2.4

Orgasm	0	6	3

Satisfaction	3.2	3.6	3.2

Pain	0	4.8	4

Total	5.3	25.2	18.3

^∗^ 6 months after implant.

^∗∗^ 12 months after explant.
